# Estimation of Instantaneous Complex Dynamics through Lyapunov Exponents: A Study on Heartbeat Dynamics

**DOI:** 10.1371/journal.pone.0105622

**Published:** 2014-08-29

**Authors:** Gaetano Valenza, Luca Citi, Riccardo Barbieri

**Affiliations:** 1 Neuroscience Statistics Research Laboratory, Department of Anesthesia, Critical Care & Pain Medicine, Harvard Medical School, Massachusetts General Hospital, Boston, Massachusetts, United States of America; and Department of Brain and Cognitive Science, Massachusetts Institute of Technology, Cambridge, Massachusetts, United States of America; 2 Research Center E. Piaggio and Department of Information Engineering, University of Pisa, Pisa, Italy; 3 School of Computer Science and Electronic Engineering, University of Essex, Colchester, United Kingdom; University of California, Merced, United States of America

## Abstract

Measures of nonlinearity and complexity, and in particular the study of Lyapunov exponents, have been increasingly used to characterize dynamical properties of a wide range of biological nonlinear systems, including cardiovascular control. In this work, we present a novel methodology able to effectively estimate the Lyapunov spectrum of a series of stochastic events in an instantaneous fashion. The paradigm relies on a novel point-process high-order nonlinear model of the event series dynamics. The long-term information is taken into account by expanding the linear, quadratic, and cubic Wiener-Volterra kernels with the orthonormal Laguerre basis functions. Applications to synthetic data such as the Hénon map and Rössler attractor, as well as two experimental heartbeat interval datasets (i.e., healthy subjects undergoing postural changes and patients with severe cardiac heart failure), focus on estimation and tracking of the Instantaneous Dominant Lyapunov Exponent (IDLE). The novel cardiovascular assessment demonstrates that our method is able to effectively and instantaneously track the nonlinear autonomic control dynamics, allowing for complexity variability estimations.

## Introduction

Hearth contractions are regarded by many scientists as the foremost example of a physiological system showing predominantly nonlinear behavior, mainly generated through integration of multiple neural signaling at the level of the sinoatrial node [1]. Accordingly, the fluctuations in the interval between consecutive heartbeats have been widely investigated as output of a nonlinear system revealing and quantifying the complexity of cardiovascular control [2]–[27].

Among all nonlinearity and complexity measures, Lyapunov exponents (LEs) have been proven to provide an important mathematical tool in characterizing dynamical properties of a nonlinear system [28]. LEs were first defined by Lyapunov [29] in order to study the stability of non-stationary solutions of ordinary differential equations and for more than fifty years they have been extensively studied in many disciplines [30]–[33]. Specifically, they refer to the average exponential rates of divergence or convergence of neighboring trajectories in the system phase space. In fact, for a system whose characteristic equations are known, there is a straightforward technique for computing the whole Lyapunov spectrum [28]. Several methods for a reliable data-driven LEs estimation, even in short time data records, have been also proposed [34], [35]. In a deterministic nonlinear system with no stochastic inputs, a positive LE reflects sensitive dependence to initial conditions and can be taken as a definition of a chaotic system [36]. Nevertheless, a small amount of noise in a limit cycle oscillation could yield a positive LE if the trajectory has regions with large slopes. In chaotic systems [37], stochasticity does not play a crucial role and their dynamics are highly dependent on the initial condition. Although it is straightforward to consider chaotic mathematical systems in which stochastic inputs are suppressed, in actual applications especially related to physiological systems, it is not possible to eliminate such inputs making the chaos assessment simply unreliable [3]. Stationary aperiodic behavior, in fact, can also arise in linear or nonlinear stochastic systems. In light of these considerations, as this work deals with (instantaneous) LEs estimation with applications on heartbeat dynamics, we do not address the issue related to the chaotic behavior of heart rate variability (HRV).

Relying on the approach suggested by Chon et al. [38] and, later, by Armoundas et al. [39], we consider the cardiovascular system both chaotic and stochastic. This concept is in agreement with current physiological knowledge, since healthy HRV dynamics can be considered/modeled as the output of a nonlinear deterministic system (the pacemaker cells of sinus node) being forced by a high-dimensional input (the activity in the nerves innervating the sinus node). Accordingly, we model the heartbeat nonlinear dynamics as a third-order Nonlinear Autoregressive (NAR) model embedded in a point process probabilistic framework. Such statistical approach allows us to estimate the LEs in an instantaneous fashion by fitting the model to the observed data and applying the Fast Orthogonal Search (FOS) algorithm [40]. Point-process theory has been widely recognized as an excellent mathematical tool to characterize the probabilistic generative mechanism of the heartbeat at each moment in time [41]. In the considered model, the intrinsically discrete, unevenly spaced heartbeat intervals are represented by a physiologically-plausible inverse-gaussian (IG) distribution. Defining the first and second-order moments of the IG distribution as function of the past heartbeat intervals (i.e., the RR intervals), it is possible to obtain an effective prediction of the next heartbeat event together with an accurate assessment of instantaneous indices of cardiovascular control. In previous studies [19], [41], [42], we demonstrated how to estimate heartbeat dynamics even in short recordings under nonlinear and non-stationary conditions using Wiener-Volterra theory for nonlinear systems identification.

### Introduction to the Instantaneous Dominant Lyapunov Exponent

In this work, the IG mean is modeled as a third-order nonlinear function of the past RR intervals. In order to perform an effective parameter estimation and retain all the historical information of events, the cubic NAR kernels of the Wiener-Volterra series are expanded using the Laguerre bases [43] leading to the definition of cubic Nonlinear Autoregressive Laguerre (NARL) model. Of note, the NARL definition includes an infinite regression of the past events with a parsimonious use of model parameters. From the NARL point-process model and Fast Orthogonal Search algorithm, we are able to estimate the complete LE spectrum at each moment in time, providing novel information concerning the complexity dynamics and its variability. Of note, to the best of our knowledge, complexity variability measures have never been estimated from instantaneous indices of complexity and could open novel perspectives on the assessment of discrete stochastic physiological systems. We present two applications on synthetic datasets (the Hénon map and Rössler attractor) and two experimental applications portraying the crucial role of the instantaneous Lyapunov Exponents in assessing autonomic changes in humans (ten heathy subjects undergoing postural changes, and fourteen patients with severe heart failure), focusing our attention on the Instantaneous Dominant Lyapunov Exponent (IDLE, 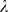), which is the first exponent of the Lyapunov spectrum. Preliminary results of these analyses have been presented in [44]–[46]. We start with a detailed, exhaustive presentation of our methodological framework through the following “Materials and Methods” section.

## Materials and Methods

### Point-Process Models of Heartbeat Dynamics

A random point process is a stochastic process whose elements are point patterns specified as a locally finite counting measure [47]. Considering the R-waves detected from the Electrocardiogram (ECG) as such events, point process theory can be used to characterize their probability of occurrence [41], [42], [48]. Mathematically, in the time domain, a simple 1-dimension point process consists of series of timestamps marking the occurrence times 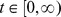 of the random events. Given a set of R-wave events 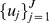, let 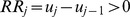 denote the 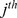 R–R interval, or equivalently, the waiting time until the next R-wave event. Assuming history dependence, the probability distribution of the waiting time 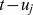 until the next R-wave event, where 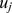 denotes the previous R-wave event occurred before time 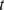, follows an inverse Gaussian (IG) model: 
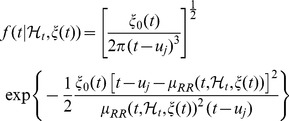
(1)where 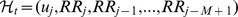 is the history of the point process, 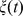 is the vector of the time-varying parameters, 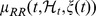 represents the first-moment statistic (mean) of the distribution, and 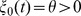 denotes the shape parameter of the IG distribution (as 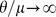, the IG distribution becomes more like a Gaussian distribution). As 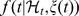 indicates the probability of having a beat at time 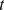 given that a previous beat has occurred at 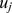, 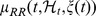 can be interpreted as signifying the average (or expected) waiting time before the next beat. We can also estimate the second-moment statistic (variance) of the IG distribution as 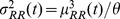. The use of an IG distribution to characterize the R-R intervals occurrences is motivated by the fact that if the rise of the membrane potential to a threshold initiating the cardiac contraction is modeled as a Wiener process with drift, then the probability density of the times between threshold crossings (the RR intervals) is indeed the inverse Gaussian distribution [41], [49]. As a matter of fact, when compared with other distributions, the IG model achieves the overall best fit in terms of smaller KS distance [42]. The instantaneous RR mean, 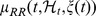, can be modeled as a generic function of the past RR values 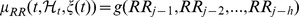, where 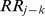 denotes the previous 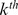 R–R interval occurred prior to the present time 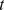.

### Nonlinear Modeling of History Dependence

The general Nonlinear Autoregressive (NAR) formulation can be written as: 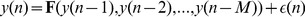
(2)where 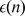 are independent, identically distributed (i.i.d.) Gaussian random variables. The expected value of 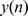 can be written as a Taylor expansion under the hypothesis of being infinitely differentiable in a neighborhood of the working point: 
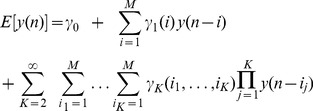
(3)


Due to the autoregressive structure of eq. 3, the system can be identified with only exact knowledge of the output data and with only few assumptions on the input data. In practice, this series will obviously be truncated at some small, finite value. Here, we represent the nonlinear cardiovascular system by taking into account up to the cubic nonlinear terms, i.e. 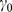, 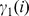, 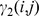, and 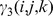. Thus, the model can be written as: 
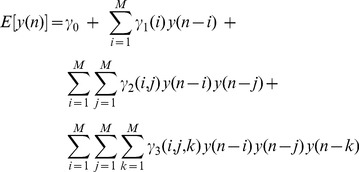
(4)where the quadratic and the cubic terms, 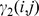 and 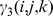, are assumed to be permutation invariant. This choice of a third order NAR system further gives robustness against the presence of measurement noise in the data [38].

#### Laguerre Expansion of the 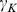 terms

An important practical limitation in modeling high-order nonlinearities using the model in eq. 4 is the high number of parameters needed to sufficiently fit the observed data. An advocated approach to solve such a limitation has been proposed by using the Laguerre functions [43], [50], [51]. Let us define the 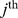-order discrete time orthonormal Laguerre function: 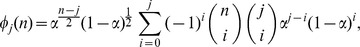
where 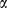 is the discrete-time Laguerre parameter (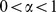) which determines the rate of exponential asymptotic decline of these functions, and 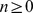. Given the Laguerre function, 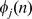, and the signal, 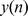, the 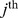-order Laguerre filter output is: 
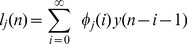
(5)


The computation of the Laguerre Filter output can be simplified by using the following recursive relation [43]: 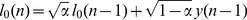
(6)

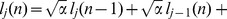
(7)

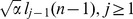
(8)


Since the 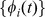 form a complete orthonormal set in functional space 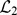, we can write [52]: 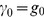
(9)

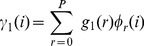
(10)

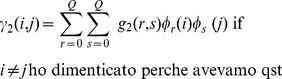
(11)

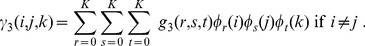
(12)


Here 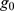, 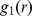, 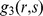 and 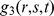 are the Laguerre coefficients. Using eq. 5 and eqs. 9–12, the model in eq. 4 for the instantaneous RR mean becomes: 
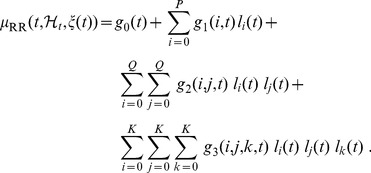
(13)hereinafter called Nonlinear Autoregressive with Laguerre expansion (NARL) model. Here, the Laguerre filter outputs are:
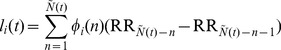
(14)with 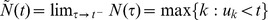 as a left continuous function.

The coefficients 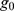,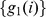, 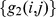, and 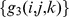 correspond to the time-varying zero-, first-, second-, and third-order NARL coefficients, respectively. When 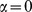 the filter output becomes 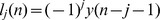 and the NARL model corresponds, apart for the sign, to the finite NAR model in eq. 4, whereas for 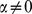 the instantaneous RR mean in terms of NAR equations is theoretically defined as follows:
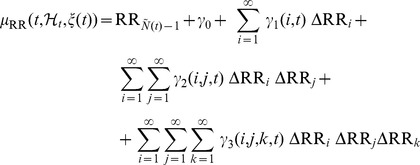
(15)where 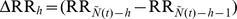. The autoregressive model is expressed in terms of the derivative RR series, rather than the original RR series, in order to allow the model to cope with highly non-stationary regimes [53].

Moreover, as 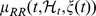 is defined in a continuous-time fashion, we can obtain an instantaneous R–R mean estimate at a very fine timescale (with an arbitrarily small bin size 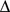), which requires no interpolation between the arrival times of two beats. Given the proposed parametric model, the nonlinear indices of the HR and HRV will be defined as a time-varying function of the parameters 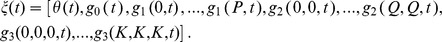


#### Parameter Estimation and Model Goodness-of-fit measures

A local maximum likelihood method [41] is used to estimate the time-varying parameter set 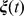. Given a local observation interval 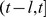 of duration 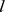, we consider a subset 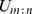 of the R-wave events, where 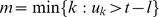 and 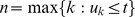. At each time 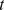, we find the parameter vector 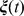 that maximizes the local log-likelihood, given the R-wave events recorded in the local observation interval: 
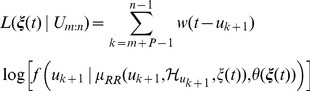
(16)where 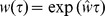 with 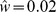 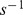, is an exponential weighting function for the local likelihood. This value has been empirically chosen by considering a range of discrete values (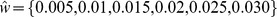), and by choosing the optimum according to KS plots goodness-of-fit analysis, as described in [41]. We use a Newton-Raphson procedure to maximize the local log likelihood in eq. 16 and compute the local maximum-likelihood estimate of 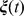. Because there is significant overlap between adjacent local likelihood intervals, we start the Newton-Raphson procedure at 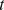 with the previous local maximum-likelihood estimate at time 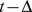 in which 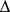 define how much the local likelihood time interval is shifted to compute the next parameter update. We determined the optimal orders 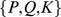 using the Akaike Information Criterion (AIC) by fitting a subset of the data using local likelihood method [41]) as well as the Kolmogorov-Smirnov (KS) statistic in the 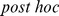 analysis. More in detail, we can compare the AIC scores and choose the parameter setup with the minimum AIC value of 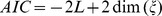 where 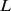 is the maximized value of the likelihood function for the estimated model, and 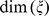 is the number of parameters in the statistical model.

It is known from point process theory [41] that the Conditional Intensity Function (CIF) 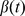 is related to the inter-event probability 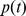 with a one-to-one relationship: 
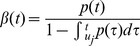
(17)


The estimated CIF is used to evaluate the goodness-of-fit of the proposed heartbeat interval point process probability model, which is based on the KS test [41].

### Instantaneous Lyapunov Exponents Estimation

The Lyapunov Exponent (LE) of a real valued function 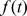 defined for 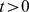 is: 
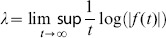
(18)


More generally, let us consider an 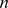-dimensional linear system in the form 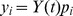, where 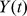 is a fundamental solution matrix with 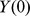 orthogonal, and 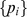 is an orthonormal basis of 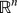. Then, the sum of the corresponding 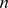 Lyapunov Exponents (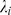) is minimized, and the orthonormal basis 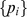 is called “normal” [54]. One of the key theoretical tools for determining LEs is the continuous QR factorization: 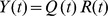 [55], [56] where 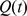 is orthogonal and 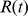 is upper triangular with positive diagonal elements 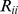, 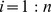. Therefore we obtain [54]–[56]: 
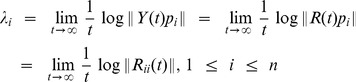
(19)


In our case, the matrix 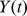 corresponds to the Jacobian of the 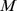-dimensional system of the NARL model parameters, where 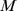 is the value of the largest model order [39]. Therefore, given the NARL model reported in eq. 13 and using eqs. 9 to 12 bringing back to the NAR framework, it is possible to obtain an 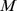-dimensional state space canonical representation.

Using the Einstein notation, we obtain: 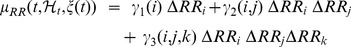
(20)


Therefore, the elements of the Jacobian can be computed as follows: 
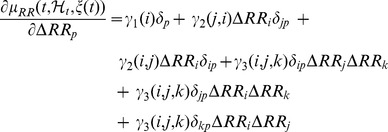
(21)and considering the symmetry properties of the NAR kernels, we finally obtain: 
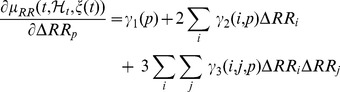
(22)


Considering 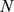 data samples, we evaluate the Jacobian over the time series, and determine the LE by means of the QR decomposition: 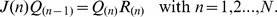



The matrix 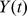 corresponds to the Jacobian of this system [39]:



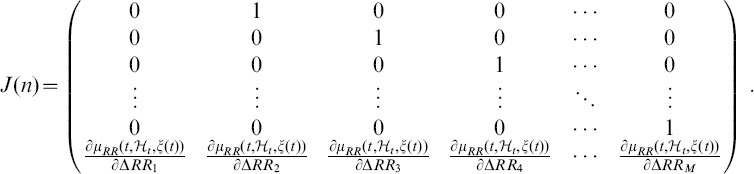
This decomposition is unique except in the case of zero diagonal elements. Then, the LEs 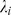 are given by 
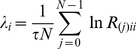
(23)where 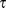 is the sampling time step. The estimation of the LEs is performed at each time 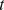 from the corresponding time-varying vector of parameters, 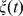. We define the first LE (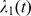) as the instantaneous dominant Lyapunov exponent (IDLE). In particular, the median IDLE (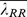) and its median absolute deviation (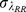) were considered as group features.

### Standard and Nonlinear Measures of Heartbeat Dynamics

In order to perform a comparison analysis with standard and nonlinear estimates of heartbeat dynamics, we also calculated the standard mean of the RR intervals (Mean RR), the root mean square of successive differences of intervals (RMSSD) and the number of successive differences of intervals which differ by more than 50 ms (pNN50% expressed as a percentage of the total number of heartbeats analyzed) [57]. Referring to morphological patterns of HRV, the triangular index is obtained as the integral of the histogram (i.e. total number of RR intervals) divided by the height of the histogram which depends on the selected bin width [57]. Moreover, we performed the estimation of the dominant Lyapunov exponent according to the algorithm described by Wolf et al. [34] (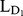) and Rosenstein et al. [58] (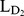). Both algorithms are suitably applied to experimental noisy data. Finally, other nonlinear measures such as the approximate entropy (ApEn) [59], Sample Entropy [60], and the Detrended Fluctuation Analysis (DFA) [61] were evaluated.

## Experimental Data

### Synthetic Data

Before reporting the implementation of the synthetic datasets, it is important to clarify some important differences from standard definitions that are introduced by our methodology. In fact, standard nonlinear systems such as the Hénon Map and Rössler Attractor are intrinsically defined in the continuos time domain, whereas our methodology deals with stochastic point processes which are a sequence of events. Moreover, additive noise terms have to be considered as well. Therefore, starting from the canonical equations of each nonlinear system, we slightly modify the system equations by adding a noise term. Then, the output of the system is taken as an input of an integrate-and-fire system. The output of such an integrate-and-fire system constitutes the series modeled by the proposed cubic NARL model within the point-process framework.

#### Hénon Map

In order to test the efficiency of the proposed cubic NARL model in tracking the complexity of a synthetic stochastic series through the IDLE index, we simulated a modified version of the well-known chaotic Hénon Map as suggested in [39]. Such a complex system, in which stochastic terms are also considered, is governed by the following differential equations: 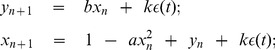
(24)


**Figure 1:**
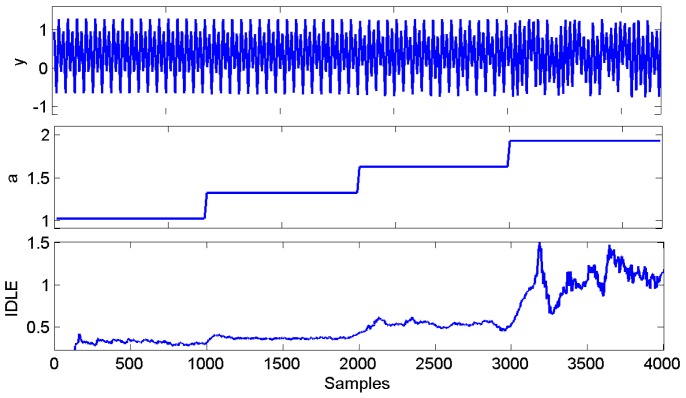
Simulation results using the Hénon equations. (Top) Synthetic interval series from the Hénon map, (Middle) the 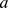 stepping, and (Bottom) IDLE averaged estimate from 100 realizations using NARL model with noise level of 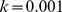.

The time series 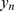 were taken into account fixing 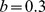. The term 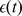 is an independent identically distributed Gaussian random variable with zero mean and standard deviation of 1, which is modulated by the coefficient 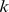. The coefficient 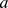 is taken as a time-varying variable from 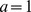 to 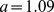 with step size 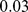. Note that the value 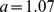, in a purely deterministic domain, corresponds to the transition between the non-chaotic and chaotic regime. A total of 100 realizations the Hénon Map series were simulated, each of which was comprised of 4000 data points with 1000 samples for each of the four 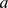-values. A realization of the simulated time series is illustrated in Fig. 1 along with the 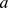-values and the corresponding IDLE results.

#### Rössler Attractor

As a further validation, we simulated a modified version of the well-known chaotic Rössler time series (see previous work [19]). Such a complex system, in which stochastic terms are also considered, is governed by the following differential equations: 
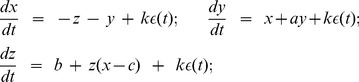
(25)


**Figure 2:**
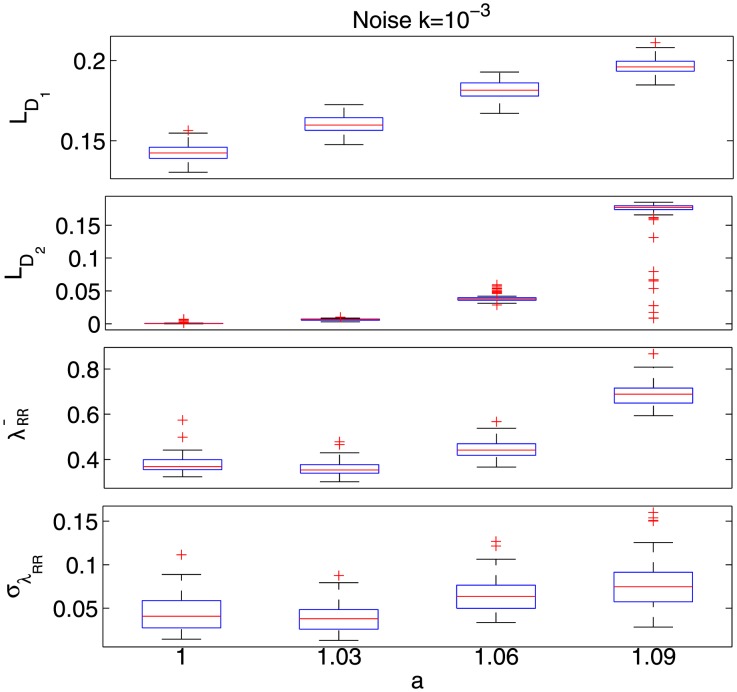
Simulation results using the Rössler equations. (Top) Synthetic interval series from the Rössler system, (Middle) the 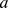 stepping, and (Bottom) IDLE estimates using NARL model. The dotted vertical line indicates the transition between the non-chaotic and chaotic regime.

The time series were implemented with sampling time of 0.01 using the Runge-Kutta integration and fixing 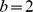, and 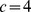. The term 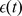 is an independent identically distributed Gaussian random variable with zero mean and standard deviation of 0.01. The coefficient 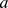 is taken as a time-varying variable from 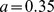 to 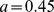 with step size 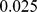. Note that the value 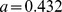, in a purely deterministic domain, corresponds to the transition between the non-chaotic and chaotic regime. A total of 75000 data points were generated with 15000 samples for each of the five 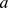-values. The simulated time series is illustrated in Fig. 2 along with the 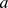-values and the corresponding results on the IDLE. The IDLE transitions to positive values reflect the simulated switch to chaotic behavior.

### Experimental Data

In order to validate the proposed algorithms performance as related to actual cardiovascular dynamics, we have considered two experimental datasets. Since the experimental protocols are fully described in [19], [41] in this paragraph we provide only briefly descriptions of the two datasets.

#### Postural Changes

In order to validate the proposed algorithms performance as related to actual cardiovascular dynamics, we studied the complexity of RR interval series recorded from 10 healthy subjects for the study of cardiovascular and autonomic regulation undergoing a tilt-table protocol. This choice is motivated by the high presence of nonlinearity and non-stationarity in such time series. In this study, a subject lying horizontally in a supine position is then actively or passively tilted to the vertical position. The rest (supine) and upright sessions alternated six times with three possible modality of transition: active stand-up, slow and fast passive tilt. A single-lead ECG was continuously recorded for each subject during the study, and the RR intervals were extracted using a curve length-based QRS detection algorithm [62]. Further details on the experimental protocol can be found in [19], [41]. The study was conducted at the Massachusetts Institute of Technology (MIT) General Clinical Research Center (GCRC) and was approved by the MIT Institutional Review Board and the GCRC Scientific Advisory Committee. Patient records/information was anonymized and de-identified prior to analysis.

#### Congestive Heart Failure

The second heartbeat dataset was constituted from data gathered from Congestive Heart Failure (CHF) and reference healthy subjects on a public source: Physionet (http://www.physionet.org/) [63]. The RR time series were recorded from 14 CHF patients (from 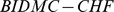 Database) as well as 16 healthy subjects (from 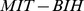 Normal Sinus Rhythm Database). Each RR time series was artifact-free (upon human's visual inspection and artifact rejection) and lasted about 50 min (small segments of the original over longer recordings). These recordings have been taken as landmark for studying complex heartbeat interval dynamics [3], [8].

## Results

### Instantaneous Complex Dynamics on Synthetic Data

#### Hénon Map

**Figure 3:**
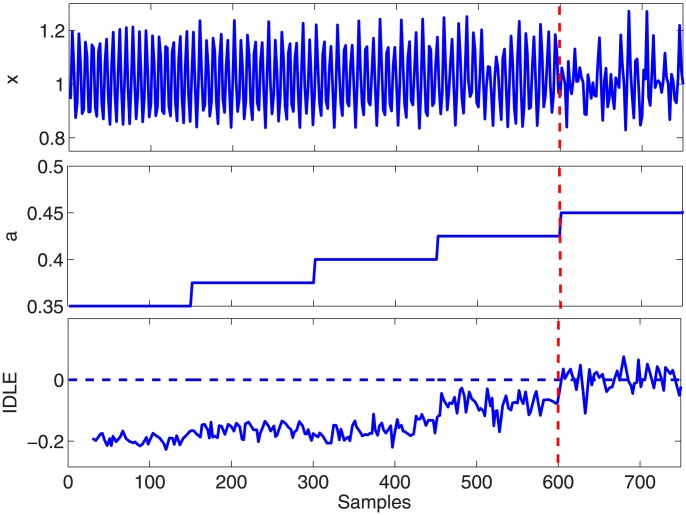
Box plots of standard and proposed Lyapunov estimations performed on the Hénon map among the *α*−stepping with noise level of *k* = 0.001.

We performed the IDLE estimation by fitting the NARL model on 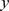 series from the modified stochastic Hénon Map time series (see eq. 24). The series were generated a hundred times for each of the four considered noise levels 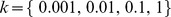. For 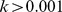, a further constrain of 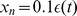 was imposed as 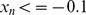 or 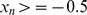 in order to prevent the system to become unstable. The model orders were set as 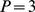, 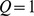, 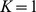, and 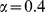 were chosen by preliminary KS plots goodness-of-fit analysis, according to [41]. The simulated time series along with the resulted IDLE series are shown in Fig. 1, whereas the corresponding box plots are shown in Fig. 3 in terms of IDLE median (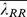) and its median absolute deviation 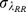. The proposed IDLE is able to track the complexity variation at each moment in time. As a matter of fact, the IDLE goes increasingly high from the non-chaotic behavior to the chaotic one. Remarkably, the non-chaos–chaos transition is instantaneously detected, although a significant oscillatory dynamics is present in the chaotic region. The related IDLE values are reported in Table 1 along with standard estimates of the dominant Lyapunov exponents. A non-parametric statistical analysis has been performed in order to quantify the differences between the considered 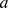-values for each of the considered noise level.

**Table 1:** IDLE Results from the Hénon map Synthetic Dataset.

a-values	1	1.03	1.06	1.09	
*Noise k = 0.001*					p-value
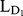	0.1424 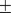 0.0035	0.1598 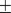 0.0041	0.1814 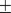 0.0040	0.1960 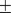 0.0030	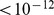
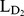	0.0004 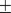 0.0002	0.0064 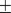 0.0009	0.0377 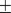 0.0019	0.1772 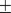 0.0030	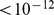
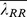	0.3679 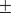 0.0195	0.3530 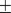 0.0179	0.4410 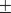 0.0268	0.6885 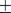 0.0349	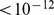
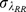	0.0407 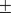 0.0151	0.0379 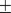 0.0112	0.0636 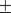 0.0130	0.0747 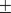 0.0170	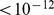
*Noise k = 0.01*					p-value
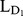	2.5488 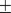 0.0251	2.5552 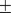 0.0387	2.5537 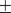 0.0280	2.5573 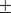 0.0238	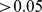
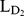	0.3038 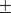 0.0070	0.3049 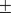 0.0077	0.3037 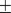 0.0085	0.3033 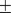 0.0083	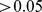
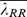	1.2240 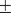 0.3232	1.5006 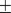 0.4380	1.4822 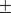 0.5034	1.4962 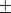 0.5237	<5_*_10^−4^
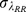	0.4511 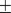 0.1559	0.6884 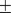 0.2008	0.5780 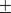 0.2476	0.7137 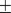 0.2830	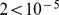
*Noise k = 0.1*					p-value
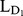	2.6704 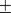 0.0265	2.6710 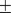 0.0283	2.6756 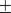 0.0306	2.6719 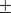 0.0350	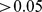
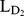	0.2069 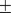 0.0997	0.2161 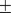 0.1221	0.2320 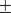 0.1334	0.2334 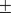 0.1459	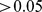
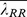	0.1180 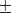 0.0517	0.1383 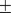 0.0565	0.1319 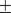 0.0487	0.1566 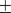 0.0468	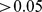
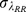	0.1348 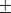 0.0376	0.1398 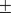 0.0314	0.1360 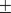 0.0378	0.1369 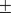 0.0247	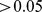
*Noise k = 1*					p-value
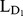	0.2949 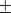 0.0033	0.2953 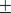 0.0031	0.2949 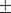 0.0031	0.2947 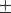 0.0023	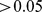
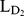	0.0023 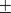 0.0022	0.0015 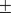 0.0015	0.0011 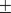 0.0011	0.0017 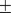 0.0016	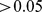
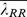	−0.1032 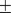 0.0104	−0.1018 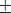 0.0107	−0.0998 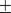 0.0098	−0.0993 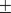 0.0119	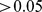
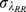	0.0318 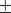 0.0056	0.0311 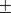 0.0071	0.0337 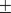 0.0066	0.0319 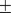 0.0071	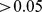

Considering the noise level 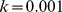, the Kruskal-Wallis test reveals significant differences (p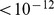) for both the standard Lyapunov estimates and the proposed 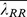 and complexity variability index 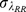. In this case, the Dunn test for multiple comparison, which considers a Tukey-Kramer correction, shows that each group of standard estimates of 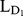 and 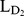 having coherent 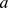 parameter are different with all the other groups (p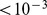), whereas coherent a-values of 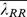 are different with all the other groups (p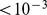) except for 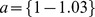 (p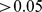).

Considering the noise level 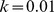, the Kruskal-Wallis test reveals significant differences (p<5_*_10^−4^) only for the proposed 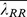 and complexity variability index 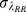. In this case, the Dunn test for multiple comparison, which considers a Tukey-Kramer correction, shows that coherent 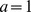 values are different with all the other groups (p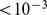), with 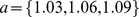 equal between each other (p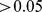). For noise level 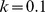 and 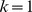, the difference between the a-values are not revealed by standard and proposed dominant Lyapunov estimates.

#### Rössler System

We performed the IDLE estimation by fitting the NARL model on the 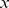 series from the modified stochastic Rössler time series (see eq. 25). The model orders were set as 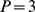, 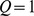, 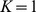, and 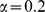 were chosen by preliminary KS plots goodness-of-fit analysis, according to [41]. The simulated time series along with the resulted IDLE series are shown in Fig. 2. Clearly, the proposed IDLE is able to track the complexity variation at each moment in time. As a matter of fact, the IDLE goes increasingly high from the non-chaotic behavior to the chaotic one. Remarkably, the non-chaos–chaos transition is instantaneously detected, although a significant oscillatory dynamics is present in the chaotic region. Intervals expressed as median 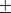 M.A.D. are as follows: 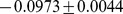 for 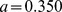, 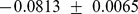 for 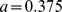, 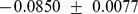 for 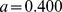, 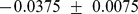 for 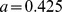, 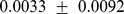 for 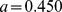. A non-parametric statistical analysis has been performed in order to quantify the differences between the considered a-values. The Kruskal-Wallis test reveals significant differences (p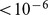) and the Dunn test for multiple comparison, which considers a Tukey-Kramer correction, shows that each IDLE group having coherent a-values is different with all the other group (p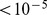) except for 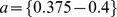 (p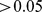).

### Instantaneous Complex Dynamics on Postural Changes

**Figure 4:**
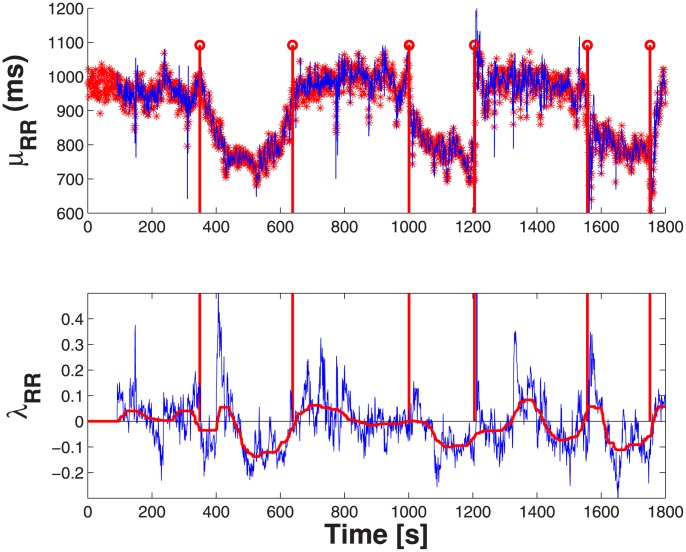
Instantaneous heartbeat statistics computed from a representative subject (subject 1) from the tilt-table protocol. In the top panel, the estimated 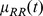 is superimposed on the recorded RR series. In the bottom panel, the instantaneous averaged IDLE is superimposed on the original IDLE.

**Figure 5:**
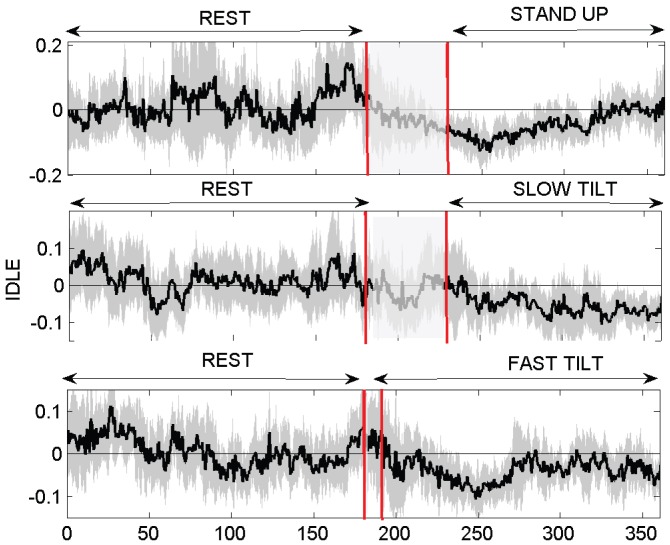
IDLE dynamics averaged for all 10 subjects. The vertical red lines indicate the transition from the supine to the upright position after stand up (top panel), after slow tilt (middle panel), and after fast tilt (bottom panel).

Before estimating the IDLE from the experimental datasets, we first considered a specific time-domain method [64] for testing the presence of nonlinearity in the heartbeat intervals. The null hypothesis of the test states that the given time series is linear. In the considered recordings, we restricted the test to short-term dependence by setting the number of laps 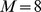, and a total of 500 bootstrap replications. Concerning the RR series gathered during postural changes, the nonlinearity test shows that the level of nonlinearity of the considered RR intervals is statistically significant for all the considered subjects but one (see Table 2. As also shown in Table 2, the NARL modeling always gives a good model fit, with KS distance 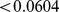 in all cases. Specifically, concerning the three experimental sessions, i.e. stand-up, slow-tilt, and fast-tilt, a decrease of the IDLE with respect to the relative rest condition is shown in 25 out of 30 epochs. In particular, in the fast-tilt condition the decrease happens for all subjects, and is more significant than stand-up and slow-tilt. Group statistics of standard and proposed instantaneous measures are shown in Table 3, whose inter-subject analysis was performed using a non-parametric rank-sum test. Results on the proposed IDLE show a non-significant statistical difference between the stand-up epochs and their relative rest epochs (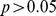) and a significant difference for the slow-tilt epochs (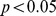). The highest significance was found comparing the fast-tilt epochs with their relative rest (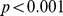). These trends are confirmed by the standard DLE estimation according to the Rosenstein et al. [58] technique, whereas the one suggested by Wolf et al. [34] did not show such significant differences. IDLE dynamics for one representative subject are shown in Fig. 4, whereas the averaged IDLEs for all 10 subjects are shown in Fig. 5, providing a clear portrayal of how different postural stimuli elicit different changes in the dynamic signatures of complexity. Concerning other standard and instantaneous indices, we report significant differences on three session on 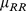, ApEn, and SampEn, whereas RMSSD and pNN50% showed significant differences during the slow and fast tilt sessions.

**Table 2:** Median and MAD of IDLE evaluated in the Tilt-Table Experimental Dataset.

Subject	P-Value	KS dist.	Rest	Stend-Up	Rest	Slow Tilt	Rest	Fast Tilt
1	0.0320	0.0458	0.0264 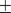 0.0298	0.0108 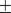 0.077	0.0304 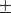 0.0466	−0.0340 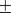 0.0514	0.0518 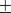 0.0227	−0.1165 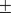 0.0326
2	0.0340	0.0603	0.1469 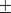 0.1712	0.0120 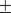 0.1794	0.2534 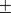 0.1007	0.0050 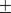 0.1372	0.2226 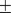 0.0988	0.0075 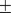 0.0515
3	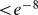	0.0355	0.0435 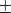 0.0552	0.0207 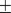 0.0232	0.0439 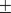 0.0745	−0.0200 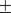 0.0423	−0.0222 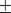 0.0662	−0.0313 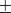 0.0574
4	0.0300	0.0227	−0.0551 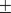 0.0150	−0.0550 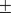 0.0185	0.0557 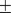 0.0679	−0.0585 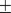 0.0315	0.0649 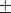 0.0785	0.0084 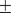 0.0365
5	0.0220	0.0451	−0.0719 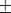 0.0613	−0.0578 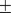 0.0547	−0.0045 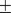 0.0441	−0.0403 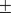 0.0355	−0.0137 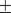 0.0595	−0.0388 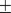 0.0226
6	0.0020	0.0409	0.0520 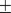 0.0894	−0.0665 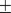 0.0368	−0.0032 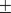 0.0692	−0.0734 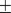 0.0442	−0.0007 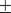 0.0786	−0.0362 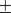 0.0303
7	0.0020	0.0458	0.0339 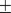 0.0448	−0.0346 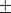 0.0589	0.0278 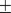 0.0343	−0.0665 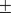 0.0341	0.0969 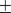 0.0406	−0.0258 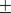 0.1049
8	0.0760	0.0408	−0.0352 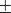 0.0467	−0.0103 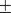 0.0668	−0.0159 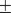 0.0571	−0.0783 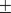 0.0405	0.0093 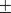 0.0518	−0.0612 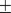 0.0375
9	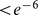	0.0571	−0.0079 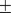 0.048	−0.0033 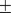 0.0703	−0.0217 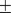 0.0365	0.0216 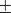 0.0391	0.0058 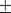 0.0394	−0.0042 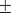 0.0145
10	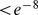	0.0572	0.6521 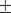 0.4771	0.0324 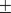 0.0830	0.2316 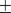 0.1737	−0.0052 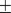 0.0650	0.2662 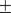 0.1708	0.0384 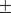 0.1653

P-values are obtained from the nonlinearity test.

**Table 3:** Group Statistics of Standard and Instantaneous Heartbeat Dynamics Measures from the Tilt-Table Experimental Dataset.

Feature	Rest	Stand-up	p-value	Rest	Slow Tilt	p-value	Rest	Fast Tilt p-value	
*Standard and Instantaneous Time Domain Measures of HRV*									
Mean RR (ms)	910.94 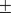 123.08	781.92 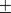 55.96	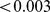	871.86 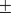 74.27	772.82 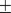 46.10	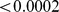	860.50 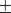 80.47	774.66 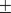 44.34	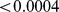
RMSSD	0.0279 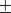 0.0123	0.0202 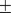 0.0055	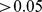	0.0325 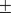 0.0127	0.0202 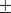 0.0048	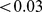	0.0296 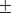 0.0127	0.0200 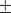 0.0042	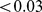
pNN50%	6.4335 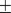 6.4335	2.3472 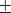 2.3472	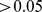	10.0744 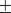 9.4524	1.6860 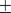 1.5708	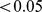	7.6677 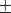 7.2816	2.0339 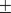 2.0339	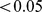
HRV Triangular Index	4.1644 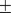 0.6832	3.9065 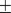 0.4754	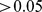	3.3012 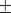 0.5142	3.5347 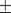 0.3497	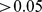	3.6429 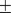 0.9743	4.3333 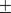 0.6352	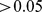
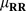 (ms)	915.10 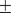 122.16	769.77 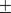 78.22	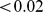	879.60 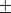 74.80	773.23 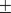 62.29	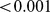	890.17 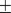 95.18	776.92 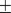 57.40	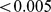
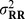 ( 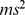 )	394.15 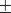 319.26	233.09 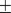 139.09	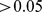	435.36 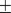 237.42	208.07 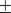 112.84	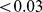	440.76 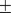 302.71	220.71 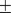 108.79	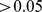
*Standard and Instantaneous Nonlinear Measures of HRV*									
ApEn	1.122 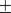 0.055	0.944 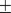 0.079	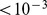	1.167 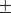 0.091	0.927 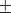 0.125	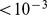	1.087 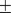 0.116	0.964 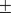 0.072	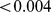
SampEn	1.501 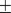 0.192	1.243 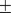 0.245	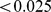	1.495 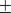 0.173	0.900 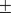 0.247	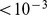	1.320 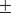 0.247	1.197 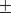 0.233	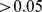
DFA- 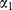	0.9806 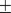 0.1039	0.9968 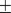 0.1624	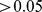	0.9892 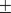 0.0952	1.2128 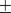 0.1126	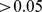	0.9788 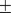 0.0990	1.0137 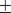 0.1425	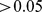
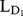	0.0128 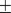 0.0014	0.0125 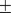 0.0013	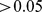	0.0133 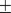 0.0017	0.0135 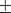 0.0015	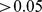	0.0137 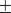 0.0013	0.0128 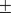 0.0008	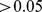
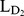	0.0029 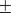 0.0005	0.0028 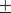 0.0005	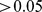	0.0037 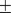 0.0006	0.0024 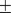 0.0004	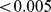	0.0033 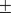 0.0006	0.0020 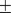 0.0007	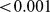
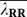	−0.0128 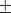 0.0480	−0.0390 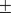 0.0330	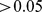	0.0205 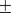 0.0372	−0.0491 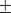 0.0236	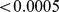	−0.0022 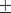 0.0332	−0.0404 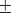 0.0232	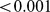
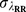	0.0585 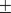 0.0213	0.0652 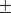 0.0178	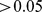	0.0693 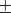 0.0098	0.0582 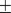 0.0113	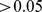	0.0617 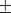 0.0131	0.0500 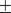 0.0144	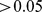

**Figure 6:**
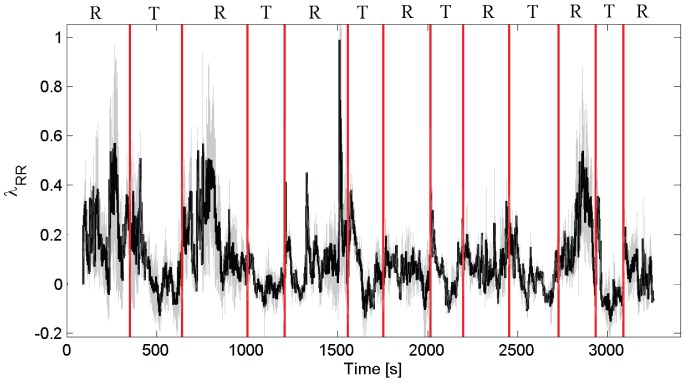
IDLE dynamics averaged for 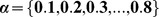 from a representative subject undergoing postural changes. Resting state (R) sessions during supine position alternate with upright session after tilt (T). The bold line and gray area indicate the IDLE median and MAD, respectively. Averaged values for all subjects are shown in Table 4.

Using this dataset, we further evaluate the effect of the Laguerre parameter 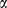 on the IDLE estimates. Tracking for values 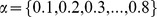 from a representative subject undergoing postural changes are shown in Fig. 6. Indeed, the IDLE estimates are affected by the choice of the Laguerre parameter 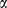. However, such a variability is significantly less than the variability of the IDLE dynamics within session. As a matter of fact, quantitative results reported in Table 4 show that these differences are associated to a p-value less than 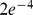 for each experimental session.

**Table 4:** IDLE Variability evaluated through 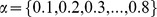 and within each session of the postural changes protocol.

Session	Through 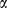 -values	Within Session	p-Value
Resting State	0.0117 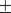 0.0044	0.0585 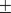 0.0213	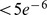
Stand-Up	0.0125 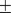 0.0082	0.0652 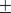 0.0178	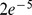
Resting State	0.0170 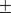 0.0240	0.0693 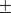 0.0098	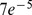
Slow Tilt	0.0119 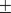 0.0128	0.0582 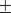 0.0113	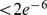
Resting State	0.0167 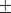 0.0192	0.0617 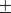 0.0131	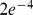
Fast Tilt	0.0119 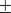 0.0128	0.0500 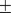 0.0144	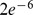

P-values are obtained from the Mann-Whitney test with null hypothesis of equal medians between the two groups. Values are expressed as 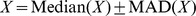.

Finally, we report further results on the nonlinearity test separately performed for each of the experimental session, instead of the whole recordings (see Table 2). As a result, under the null hypothesis of linearity according to the time-domain method described in [64] for testing the presence of nonlinearity in the heartbeat intervals, the 49.12% of the resting state (28/57 sessions) were associated to a significant p-value 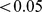, along with the 44.4% of the stand-up (8/18 sessions) protocol, the 20% of the slow-tilt (4/20 sessions) protocol, the 10.53% of the fast-tilt (2/19 sessions) protocol.

### Instantaneous Complex Dynamics on CHF patients

The results of the second experimental dataset (on CHF) are shown in Table 5. According to the nonlinearity test, 15 out of 16 RR time series from the healthy subjects showed significant nonlinearity (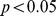), whereas in the CHF group, 6 out of 14 RR time series failed to reach significance (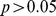). The fact that a lower degree of nonlinearity was found in the CHF patients suggests that pathological conditions might reduce the nonlinearity in the heartbeat interval series, which is also consistent with previous finding that a healthy heartbeat presents more pronounced nonlinear dynamics [3], [6], [8], [65]. Table 5 also demonstrates that the NARL model well fit both pathological and healthy heartbeat series with KS distance 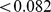 in all cases. Results averaged among groups are reported in Table 6. We report that standard and instantaneous time domain measures are able to discern the two groups with high statistical significance (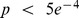). On the other hand, comparing the standard and proposed instantaneous complexity measures, only the DFA-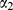 and the complexity variability 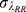 are able to provide significant discrimination capability between the two populations with 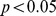.

**Table 5:** Results from the CHF-Healthy Experimental Dataset.

Subject	Group	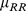 (ms)	p-value	KS dist.	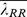	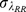
01	CHF	995.4 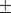 26.4	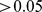	0.0445	0.2268	0.1123
03	CHF	910.25 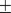 28.9	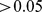	0.0552	0.2165	0.1257
04	CHF	603.09 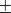 22.7	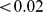	0.0456	0.0676	0.0896
05	CHF	655.6 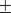 13.3	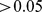	0.0297	−0.0114	0.0507
06	CHF	637.4 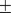 15.9	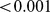	0.0802	0.0757	0.0659
07	CHF	778.0 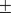 7.3	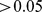	0.0363	0.0762	0.0793
08	CHF	800.1 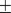 14.1	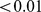	0.0357	−0.0965	0.0327
09	CHF	602.5 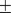 5.4	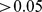	0.0305	−0.0121	0.0555
10	CHF	486.9 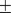 6.9	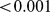	0.0329	−0.0622	0.0444
11	CHF	685.1 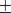 16.0	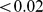	0.0354	−0.0014	0.0578
12	CHF	722.8 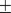 27.2	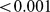	0.0326	−0.0798	0.0380
13	CHF	619.7 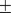 5.1	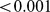	0.0386	0.0550	0.0655
14	CHF	837.7 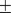 23.4	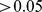	0.0367	0.0041	0.0613
15	CHF	652.15 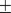 20.6	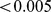	0.0265	−0.0322	0.0550
16265	healthy	1023.9 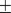 38.9	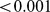	0.0527	0.0162	0.0438
16272	healthy	924.7 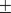 30.6	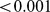	0.0539	−0.0304	0.0557
16273	healthy	1046.2 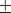 68.7	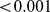	0.0764	0.0998	0.0543
16420	healthy	849.9 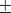 39.2	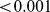	0.0394	0.0107	0.0410
16483	healthy	818.5 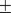 24.9	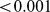	0.0336	−0.0254	0.0396
16539	healthy	831.5 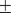 47.9	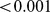	0.0592	0.0736	0.0485
16773	healthy	1238.9 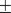 74.7	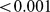	0.0819	0.0842	0.0613
16786	healthy	945.1 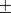 37.0	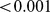	0.0503	0.0226	0.0527
16795	healthy	889.4 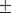 61.8	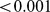	0.0442	−0.0214	0.0462
17052	healthy	939.8 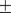 33.3	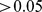	0.0487	0.0682	0.0819
17453	healthy	816.6 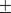 31.9	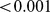	0.0416	0.0310	0.0466
18177	healthy	639.3 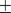 25.7	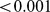	0.0267	0.0058	0.0608
18184	healthy	831.2 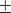 31.7	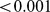	0.0392	−0.0373	0.0438
19090	healthy	993.54 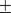 41.0	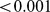	0.0565	−0.0101	0.0393
19140	healthy	849.1 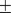 49.9	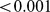	0.0353	0.0459	0.0513
19830	healthy	821.2 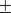 29.4	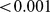	0.0452	−0.0795	0.0332

P-values are obtained from the nonlinearity test.

**Table 6:** Group Statistics of Standard and Instantaneous Heartbeat Dynamics Measures from CHF-Healthy Experimental Dataset.

	CHF (n = 14)	Healthy (n = 16)	p-value
*Standard and Instantaneous Time Domain Measures of HRV*			
Mean RR (ms)	669.73 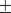 68.66	855.74 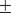 56.14	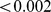
RMSSD	0.0121 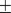 0.0036	0.0432 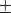 0.0145	<4 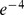
pNN50%	0.2357 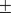 0.2246	21.5406 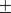 15.4908	<1 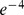
HRV Triangular Index	2.9551 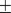 0.5769	2.5628 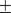 0.3593	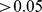
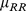 (ms)	671.55 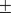 69.6	864.7 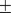 53.3	<4 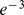
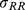 (ms)	8.31 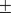 2.2	24.7 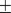 7.0	<5 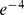
*Standard and Instantaneous Nonlinear Measures of HRV*			
ApEn	1.2130 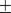 0.1032	1.2177 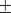 0.1066	>0.05
SampEn	1.5670 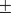 0.2690	1.4092 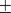 0.1522	>0.05
DFA- 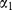	0.8498 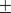 0.2191	1.0820 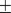 0.1467	>0.05
DFA- 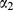	1.1552 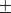 0.1335	0.9286 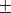 0.0544	<0.05
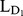	0.0167 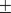 0.0025	0.0165 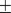 0.0012	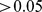
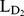	0.0029 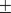 0.0008	0.0033 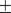 0.0005	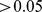
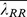	0.0014 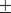 0.0649	0.0135 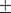 0.0368	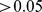
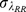	0.0595 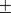 0.0120	0.0476 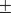 0.0066	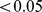

P-values are obtained from the Mann-Whitney test with null hypothesis of equal medians between the CHF and healthy subject groups. Values are expressed as 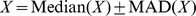.

## Discussion and Conclusion

### Novelties and Impact of the proposed Methodology

We presented a novel methodology able to instantaneously characterize the complex nonlinear dynamics of a stochastic series of events by using the LEs. The proposed approach relies on the previous literature for the LEs mathematical definition [38], [39] and is embedded in a novel IG-based point-process nonlinear framework defined through a third-order Wiener-Volterra representation, thus advancing on the previous models [19]. As a consequence, the novel instantaneous LEs definition is able to provide a reliable complexity measure tool to examine the unevenly spaced events at very high temporal resolutions, without resorting to any interpolation method. Moreover, goodness of fit measures such as KS distance and autocorrelation plots quantitatively allow to verify the model fit as well as to choose the proper model order, which represents another open issue of current parametric approaches.

The effective procedure for the time-varying parameter identification is ensured by the combined use of the discrete-time Laguerre expansions for the Wiener-Volterra terms and local maximum likelihood method. In particular, expanding the Volterra terms with the orthonormal Laguerre bases requires a reduced number of parameters to retain the information of all the past events. The nonlinear regression is further performed on the derivative series to better account for nonstationarity [53]. Importantly, unlike other methods that might require large sample size, our method is potentially useful to perform complexity measures in short recordings of the signals of interest.

Importantly, the proposed measures also allows for the study of the *complexity variability*, i.e., the analysis of complex systems referring to the fluctuations in complexity instead of analysis of central tendency. Within the proposed framework, it is always possible to incorporate physiological covariates (such as respiration or blood pressure measures) and produce further instantaneous indices from their dynamic cross spectrum and cross bispectrum [48]. Unlike other paradigms for estimating nonlinearity indices developed in the literature [7], [59], [66], [67], our method is formulated within a probabilistic framework specifically developed for point-process observations (e.g. RR intervals), which already produced important nonlinear quantifiers for autonomic assessment, based on second- and third-order statistics (instantaneous spectrum and bispectrum) [19]. Most other nonlinearity indices are derived from non-parametric models, whereas our model is purely parametric and the analytically derived indices can be evaluated in a dynamic and instantaneous fashion. We believe these strengths enable our method as a useful tool for assessing nonlinear dynamics of heartbeat intervals in a non-stationary environment.

### Study of the Instantaneous Cardiovascular Complex Dynamics

The novel IDLE index was evaluated in both synthetic and experimental heartbeat series. Estimations on the synthetic dataset were performed on a stochastic version of the well-known chaotic Hénon Map and Rössler attractor. The use of such a modified version of the Hénon Map and Rössler system alongside the obtained IDLE results need to be discussed. We are aware that in purely deterministic Rössler equations the first LE should be zero in the non-chaotic region, whereas it should be increasingly positive in the chaotic region. However, the IDLE results show slightly negative values in the non-chaotic region. Such a behavior may be ascribed to the stochastic input and to the additional integrate-and-fire step which affect the estimation of all complexity measures, including LEs. To this extent, in order to further investigate the effect of noise, results from the Hénon Map equations were gathered as a function of the noise level. We demonstrate that, for small amount of noise (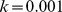), standard and instantaneous estimates of the dominant Lyapunov exponent achieve similar results. However, considering Hénon Map dynamics with 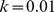, the IDLE is exclusively able to discern the different behavior of the nonlinear system. Of note, for 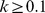, the noise has an amplitude comparable with the output of the system, thus destroying the different behaviors among the 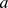-values.

The use of the Laguerre expansion of the Wiener-Volterra kernels was also investigated through experimental analysis. As the zero-order Laguerre basis is an exponential function, the IDLE estimates present a mild dependence on the 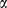 value of the Laguerre functions. Nevertheless, we demonstrated that the actual information needed to characterize the experimental sessions, i.e., the variability within each session, is significantly higher than the variability among all the 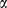 values. Anyway, using the hereby proposed approach we clearly demonstrate the ability of the IDLE in tracking the system complexity in an instantaneous fashion. The IDLE, in fact, becomes higher when the simulated system switches from non-chaotic to chaotic behavior (see Fig. 2). In all applications, an IG probability model was used as a stochastic version of the widely-applied deterministic integrate-and-fire models used to simulate heartbeats. Regarding the experimental datasets, we demonstrated that our approach is useful in characterizing the inherent nonlinearity of the cardiovascular system. For the first time, tracking complexity by instantaneous Lyapunov Exponents was performed and evaluated during postural changes. During the resting condition the cardiovascular and autonomic nervous system are more sensitive to the initial conditions (positive IDLE), whereas a more regular dynamics (negative IDLE values) appear during the tilt phases (see Fig. 4). These results are in agreement with previous findings that complex vagally-driven dynamics are blunted under sympathetic drive [68] and with more recent reports on loss of complexity during states of arousal [20]). Our instantaneous measures also confirm that the instantaneous complexity reflects instantaneous autonomic nervous system (ANS) control on the cardiovascular dynamics. We have shown that tracking ANS complexity on healthy subjects undergoing postural changes not only confirms previous results [69], [70], but further improves sympathovagal assessment as elicited by different dynamic gravitational stimuli.

Our experimental findings on the nonlinearity test performed on each experimental session (resting state, stand-up, slow tilt, and fast tilt) suggest that loss of instantaneous heartbeat complexity as a function of velocity of the postural changes is reasonably due to changes in the nonlinearity of the cardiovascular system (instead of, for example, changes on the noise properties). Of note, we have previously reported that the standard HRV indices defined in the time and frequency domain are unable to distinguish the three possible modality of transition through different p-values [19].

Moreover, the novel complexity features {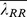; 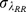} give important information in the complexity evaluation, also useful in distinguishing heartbeat dynamics coming from patients with CHF and healthy subjects. We found that pathological heartbeat dynamics are associated with increased complexity variability, providing an unique measure of complexity able to discern the CHF and healthy populations. Of note, some of the standard measures defined in the time domain have similar p-value than the point-process measures, as well as DFA-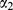 shows similar performances than the IDLE median absolute deviation. Nevertheless, we point out that we aimed at showing the performances of novel instantaneous measures of complexity based on Lyapunov exponents, providing novel insights on the complexity characterization of stochastic time-varying discrete point-process systems. In other words, although other HRV-based measures are able to discern CHF from healthy subjects, no other measures have been proposed to characterize the time-varying complexity behavior occurring in pathological vs. a healthy cardiovascular system. In particular, while confirming the results reported in the current literature (i.e., several measures of complexity are not able to characterize the CHF and healthy subjects groups), we show a novel key complexity behavior through the proposed complexity variability framework. These findings can be linked to the current literature whereby cardiovascular disorders affect complexity and variability, and may lead to serious pathological events such as heart failure [71].

Concerning other preliminary applications, the proposed IDLE methodology has been revealed as a powerful tool also in tracking the instantaneous complexity during loss of consciousness induced by anesthetic drugs [46]. Looking at the overall results shown using actual heartbeat dynamics data, it seems that the median IDLE is sensitive to changes in ANS regulation induced by orthostatic stress, while IDLE median absolute deviation is sensitive to changes in ANS regulation induced by CHF. However, this conclusion cannot be made without speculation at this time as the data used for the comparison between CHF patients and healthy subjects is related to long-term ECG monitoring during unstructured activity, whereas the data from the tilt-table experimental dataset is structured. Therefore, the observed sensitivity of the IDLE measures could be due to different physiological behavior occurring in CHF subjects or to differences related to the kind of experimental protocol. Future works are related to pursue this direction in further investigating the potential of these high-order nonlinear models in producing new real-time measures for the underlying complexity of physiological systems, and to the investigation of the instantaneous complexity, along with the baroreflex sensitivity and respiratory sinus arrhythmia, during postural changes in CHF subjects, thus allowing some conclusions on the complex physiological behavior of the cardiovascular system in CHF subjects.
